# New Tools to Study Astrocyte Ca^2+^ Signal Dynamics in Brain Networks *In Vivo*

**DOI:** 10.3389/fncel.2017.00134

**Published:** 2017-05-09

**Authors:** Gabriele Losi, Letizia Mariotti, Michele Sessolo, Giorgio Carmignoto

**Affiliations:** ^1^Neuroscience Institute, National Research Council (CNR) and Department of Biomedical Sciences, University of PadovaPadova, Italy; ^2^Division of Neurobiology, MRC Laboratory of Molecular BiologyCambridge, UK; ^3^Center for Drug Discovery & Development, Aptuit inc.Verona, Italy

**Keywords:** astrocytes, calcium signaling, optogenetics, DREADD, imaging *in vivo*

## Abstract

Sensory information processing is a fundamental operation in the brain that is based on dynamic interactions between different neuronal populations. Astrocytes, a type of glial cells, have been proposed to represent active elements of brain microcircuits that, through dynamic interactions with neurons, provide a modulatory control of neuronal network activity. Specifically, astrocytes in different brain regions have been described to respond to neuronal signals with intracellular Ca^2+^ elevations that represent a key step in the functional recruitment of astrocytes to specific brain circuits. Accumulating evidence shows that Ca^2+^ elevations regulate the release of gliotransmitters that, in turn, modulate synaptic transmission and neuronal excitability. Recent studies also provided new insights into the spatial and temporal features of astrocytic Ca^2+^ elevations revealing a surprising complexity of Ca^2+^ signal dynamics in astrocytes. Here we discuss how recently developed experimental tools such as the genetically encoded Ca^2+^ indicators (GECI), optogenetics and chemogenetics can be applied to the study of astrocytic Ca^2+^ signals in the living brain.

## Introduction

Brain function is based on complex networks composed of different, highly interacting cell populations. The activity of principal projecting neurons is locally regulated by different classes of interneurons (Markram et al., 2004; Ascoli et al., 2008; Isaacson and Scanziani, 2011) as well as by the glial cells astrocytes. These non-neuronal cells constantly interact with neurons and exert functions beyond their classical role in brain tissue homeostasis. Indeed, astrocytes sense neuronal activity and release gliotransmitters such as glutamate, ATP and D-serine, modulating synaptic transmission, controlling neural network excitability (Araque et al., 2014; Bazargani and Attwell, 2016) and contributing to neurovascular coupling mechanisms (Zonta et al., 2003; Filosa and Iddings, 2013; Howarth, 2014). Gliotransmitter release occurs through mechanisms that are only partially identified, and it is regulated by intracellular Ca^2+^ oscillations induced by different neurotransmitters (Zorec et al., 2012; Sahlender et al., 2014; Bazargani and Attwell, 2016). Accordingly, these Ca^2+^ changes represent a key step in functional neuron-astrocyte interactions. It follows that single or two-photon laser-scanning microscope Ca^2+^ imaging is a suitable approach to evaluate the activity of astrocytes from *in vitro* and *in vivo* preparations. Noteworthy, although the tools used to study neuronal Ca^2+^ signals are commonly applied also to study astrocytic Ca^2+^ signals, astrocytes exhibit peculiar properties that must be taken into careful consideration. For instance, Ca^2+^ sensitive dye choice is crucial for astrocytes as the processes, that are in contact with synapses, are nanoscopic fine lamellipodia-like structures (Rusakov, 2015). Accordingly, the Ca^2+^ signal changes associated with these structures can be more accurately monitored with genetically encoded Ca^2+^ indicators (GECI) rather than bulk-loaded classical Ca^2+^ dyes. Here we summarize the innovative techniques to study neuron-astrocyte interactive networks *in vivo*, describe advantages and limitations and discuss possible future developments in this field.

## Imaging Techniques

**Figure 1:**
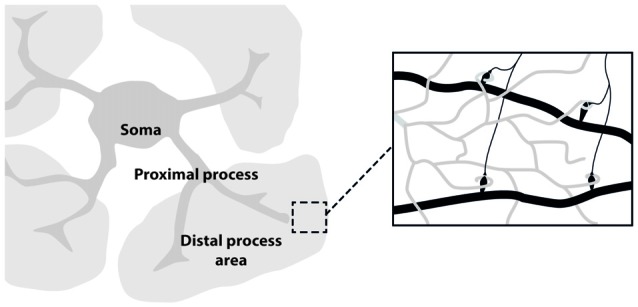
**Astrocyte main sub-structures**. Soma, thick proximal processes and fine distal processes. These latter form a mesh of ultra-thin protrusions (below optical resolution; gray) in contact with synapses (black; right inset).

The development of *in vivo* optical imaging, especially two-photon laser microscopy, is providing important information on neuronal as well as astrocytic networks in mammalian brain (Göbel and Helmchen, 2007; Ding, 2013). Although the imaging techniques used for studying astrocytes are essentially the same as those for neurons, astrocyte unique morphology and physiology must be taken into account in the choice of the proper experimental design. Astrocyte morphology comprises three major compartments: the soma, the few thick proximal processes and the nanometric, densely arborized fine distal processes (see Figure 1). Each of these compartments likely has distinctive functional properties that give rise to the extremely complex spatio-temporal Ca^2+^ dynamics observed in astrocytes. The different spatial scale and Ca^2+^ dynamics peculiar to each of these compartments set up different challenges for imaging astrocyte function. The astrocytic soma is 5–10 μm in diameter and is characterized by slow, sustained Ca^2+^ changes. Ca^2+^ elevations at the soma are not commonly induced by low levels of synaptic activity and are preferentially activated by an intense firing in the surrounding neuronal circuits (Perea and Araque, 2007). Proximal processes are typically 2–5 μm thick, about 20 μm long and are characterized by small, rapid and localized Ca^2+^ elevations that can evolve in expanded intracellular waves eventually propagating to the soma (Pasti et al., 1997; Di Castro et al., 2011; Panatier et al., 2011). Two photon-imaging studies suggest that astrocytic proximal processes can sense more finely synaptic transmission around them, possibly integrating signals from the finer distal processes that contact individual synapses. The distal processes (30–80 nm; Rusakov, 2015) appear as multiple, blurred, faint bushes tiling the entire astrocytes domain. Because of the limitations of the optical resolution, distal processes are difficult to image. Recent experiments reveal a higher frequency in Ca^2+^ events with an even faster kinetics in these processes compared to what observed in proximal processes and soma (Srinivasan et al., 2015; Poskanzer and Yuste, 2016).

Imaging of astrocyte function is boosted by the application of *in vivo* two-photon microscopy in awake behaving animals, particularly because Ca^2+^ signals in astrocytes are strongly depressed during anesthesia (Schummers et al., 2008). Refinement of cranial window implants (Goldey et al., 2014) and behavioral paradigms for head fixed animals are allowing investigation of astrocyte function in the neocortex (Perea et al., 2014; Monai et al., 2016) and astrocyte plasticity in long-term chronic preparations. Microscopes equipped with resonant scanners and piezoelectric z-drivers will allow to record from large cortical columns (in the order of millimeters, Sofroniew et al., 2016) or from the whole 3D arborization of a single astrocyte with high spatial and temporal resolutions (i.e., for resonant galvanometers from 30 Hz for 512 pixels to 60 Hz for 256 pixels; for review see Ji et al., 2016).

Although the use of longer wavelength light for two-photon compared to single-photon excitation laser-scanning microscopy has increased light penetration, two-photon imaging is essentially restricted to structures such as neocortex, olfactory bulb or cerebellar cortex, where signals from cells within 1 mm from brain surface can be visualized. The introduction of three-photon imaging promises to extend these techniques to imaging subcortical structures like the hippocampus (Horton et al., 2013). Other promising approaches include *in vivo* optical microendoscopes or microprisms that can be inserted into the brain to allow a variety of viewing angles and imaging of otherwise unreachable deep brain regions (Chia and Levene, 2009; Barretto and Schnitzer, 2012; Andermann et al., 2013; Heys et al., 2014).

Another limitation in the data analysis of astrocytic Ca^2+^ signals is the segmentation of functionally meaningful regions of interests. Astrocytic processes do not show a clear compartmentalization, such as the spines in neuronal dendrites or the synaptic boutons in axon terminals, and the identification of proximal and distal domains is often arbitrary. Various approaches have been suggested to resolve this problem (Di Castro et al., 2011; Srinivasan et al., 2015), mostly based on thresholding and segmentation of intensity projection image in domains of specific size. An interesting method would be the application to astrocytes of computational methods developed to segment recordings from large neuronal fields of view into independent regions of interest (ROIs). These algorithms are only loosely based on anatomical criteria; rather, they cluster together pixels with correlated time-courses (Pnevmatikakis et al., 2016). These methods could prove very useful for an unbiased segmentation of functional microdomains in astrocytic processes.

## Calcium Sensitive Dyes

Over the last decades fundamental insights into Ca^2+^ signal dynamics in astrocytes have been provided by organic Ca^2+^ indicator dyes. These indicators, such as Fluo-4 and Fura-2, are easily delivered to astrocytes by bulk or intracellular loading with the acetoxymethy esters (Panatier et al., 2011; Mariotti et al., 2016). Despite their easy applicability in brain slices from young animals, these indicators have many drawbacks. Indeed, loading with organic Ca^2+^ indicators of astrocytes from adult brain slices is very unsatisfactory. *In vivo* bulk-loading with Oregon Green is possible and commonly used (Takata et al., 2011), but it is time consuming and it needs intra-tissue applications that may damage the area under investigation. Furthermore this approach is inadequate for chronic applications (Roome and Kuhn, 2014). Most importantly, synthetic indicators are unsatisfactory loaded in fine astrocytic processes. The GECIs have overcome most of these limitations and represent an alternative tool for studying *in vivo* astrocyte physiology. Indeed their use provides insights into the Ca^2+^ signal dynamics at astrocyte fine processes due to a good expression also at this site. In addition, GECI can be engineered to target specific structures such as the plasma membrane or the intracellular organelles, like mitochondria, or the endoplasmic reticulum (for reviews see Takata et al., 2011; Tian et al., 2012). Indeed, a recent study used GECIs to unveil an important role of the mitochondria permeability transition pore in astrocytic Ca^2+^ signaling (Agarwal et al., 2017). While many aspects remain to be defined, important advances have been, therefore, already made in the understanding of the mechanisms at the basis of the Ca^2+^ transients in the astrocytic fine processes. GECIs consist of a Ca^2+^ binding protein linked to a conformational actuator (M13 peptide) and one or two fluorescent proteins and are extensively used for studying astrocyte physiology *in vivo*. Several tools are used to express GECIs in astrocyte: mouse lines expressing Cre recombinase under the control of specific promoters (glutamate aspartate transporter (GLAST), glial fibrillary acidic protein (GFAP) or S100 calcium-binding protein B (S100β) crossbred with GCaMP-floxed mice represent a non-invasive method to express GECIs in a subpopulation of astrocytes (Paukert et al., 2014). Other techniques include *in utero* electroporation to interrogate astrocytes during development (Gee et al., 2015; Szczurkowska et al., 2016) and adeno-associated viruses (AAV). AAV-based expression technique can be used to target astrocytes with high levels of GECIs expression in a specific brain region without the need of Cre-mice line. Recently several works have shown how GECIs can be used to study in detail Ca^2+^ activity from individual astrocyte *in vitro* and *in vivo* (Atkin et al., 2009; Shigetomi et al., 2013). GCaMP6f expression in mouse somatosensory cortex through viral vectors has provided new insights on astrocyte activity *in vivo*. Indeed GCaMP6f expressing astrocytes reveal a previously unexpected pattern of Ca^2+^ activity at the distal fine processes, i.e., microdomains (Srinivasan et al., 2015). Notably, given the complexity of, astrocytic Ca^2+^ signals, a critical step is the development of an approach to accurately measure Ca^2+^ microdomain activity. While a few algorithms have been already released (Srinivasan et al., 2015), more efforts are needed to obtain an overall valuable method of data analysis that can be shared by the scientific community. Finally, although GECIs improved the quality of Ca^2+^ activity recordings, different promoters, regulatory sequences and expression technique should be taken into account to optimize the signal-to-noise ratio and to avoid cytotoxicity.

## Selective Tools to Study Neuron-Astrocyte Crosstalk

A major issue in studying neuron-astrocyte interactions is the specificity of the stimuli used to selectively activate or inhibit one of these two cell populations without directly affecting the other. Indeed, astrocytes and neurons share most of ligand-gated receptors and astrocytes respond to a variety of neurotransmitters from glutamatergic, cholinergic and noradrenergic signaling pathways, that induce astrocytic Ca^2+^ elevations (Schummers et al., 2008; Takata et al., 2011; Navarrete et al., 2012; Paukert et al., 2014). Most *in vivo* studies on astrocytes were performed in anesthetized animals, a condition that greatly reduces spontaneous Ca^2+^ events compared to awake animals (Nimmerjahn et al., 2009; Thrane et al., 2012). It is also noteworthy that in behaving animals, astrocytes can be simultaneously recruited by the activity of different neuronal networks, making it very challenging to isolate a single component in neuron-to-astrocyte signaling. Direct astrocyte stimulation through membrane receptor-mediated signaling pathways leading to intracellular Ca^2+^ elevations in astrocytes may not be selective as these receptors are also expressed in neurons. However, with the recent development of chemogenetics and optogenetics, we have now powerful tools to interrogate selective cell populations in the living brain.

Chemogenetic approach makes use of modified G-coupled receptors, called designer receptor exclusively activated by designer drugs (DREADDs), that can be activated by selective agonists devoid of endogenous targets (for reviews see Urban and Roth, 2015; Roth, 2016; Whissell et al., 2016). DREADDs are selectively expressed in a desired cell population usually via viral vectors or using transgenic mice. The use of viral vectors has the advantage, compared to that of transgenic mouse lines, to achieve DREADD expression in the specific brain ROI, avoiding the risk of a large scale response upon agonist administration. On the other hand, it has to be taken into account that viral vectors are not completely devoid of toxicity. The most used DREADDs are the excitatory Gq- or inhibitory Gi-coupled receptors hM3Dq and hM4Di, respectively, derived from human muscarinic receptor 3 and 4. Both can be activated with the selective agonist clozapine-N-oxide (CNO), devoid of endogenous ligands, that can cross the blood brain barrier after oral or i.p. administration (Roth, 2016). This chemogenetic approach thus offers the opportunity for a noninvasive and selective activation, or inhibition, of specific cell populations. Inhibition of a selected neuronal group with DREADD approach was indeed used to oppose epileptic activity (Katzel et al., 2014; Avaliani et al., 2016). Alternatively hM3Dq can be used to selectively evoke Ca^2+^ transients in astrocytes, allowing interrogation of local neuronal circuits. This approach has been successfully used in hypothalamus astrocytes to modulate food intake (Yang et al., 2015) and in nucleus accumbens astrocytes to modulate reward and motivation (Bull et al., 2014), and reinstate cocaine seeking (Scofield et al., 2015). The timing of the response to chemogenetic approach differs substantially to other techniques such as optogenetics, which is three orders of magnitude faster (see below). DREADD activation with CNO occurs after 30 min and lasts for about 2 h (Guettier et al., 2009). This can be an option to study long lasting network effects or behaviors, but not for short lasting phenomena. Alternatively, other faster DREADD have been developed, such as a kappa-opioid receptor (KORD) coupled to a Gi-protein that has no endogenous ligands. KORD can be selectively activated by salvinorin B (SALB) that evokes shorter response than CNO on DREADDs, in the range of few minutes (Vardy et al., 2015). Therefore, the combination of multiple DREADDs provides neuroscientists with a set of choices, depending on the timing of the network activity to be studied.

The optogenetic technique was applied to mammalian cells more than 10 years ago by the group of Karl Deisseroth (Boyden et al., 2005; Fenno et al., 2011). By combining the selective expression of light-gated opsins with optical tools to stimulate, or silence, defined neuronal populations with millisecond precision (Deisseroth, 2015), optogenetics has provided a bulk of important insights into the distinct role of defined neuronal populations. Optogenetics represents an invaluable tool also to interrogate the astrocyte response to different neuronal type-specific signaling. Light stimulation of channel-rhodopsin 2 (ChR2), the most commonly used opsin, opens a cation-permeable channel that in neurons evokes action-potential firing. If ChR2 is targeted to astrocytes, light stimulation leads to Ca^2+^ elevations that trigger gliotransmitter release, including ATP or glutamate (for review see Ji and Wang, 2015). Gourine et al. revealed that in the brain stem, astrocyte Ca^2+^ transients induced by optogenetic light stimulation could control breathing through ATP release (Gourine et al., 2010). In visual cortex, instead, astrocyte optogenetic stimulation modulated neuronal activity via metabotropic glutamate receptors affecting neuronal integration of visual stimuli (Perea et al., 2014). Very recently, Archaerhodopsin (Arch), an inhibitory opsin commonly used to hyperpolarize neurons, was also used in the somatosensory cortex to evoke astrocytic Ca^2+^ elevations *in vivo* (Poskanzer and Yuste, 2016). Although the mechanism of the Ca^2+^ response to optogenetic astrocyte activation with Arch was not univocally identified, the authors reported that activated astrocytes release glutamate and modulate slow cortical oscillations *in vivo* (Poskanzer and Yuste, 2016). In conclusion, astrocyte optogenetic activation allowed a direct causal validation of previous studies reporting astrocyte regulation of slow cortical oscillations (Fellin et al., 2009; Halassa et al., 2009; Poskanzer and Yuste, 2011).

However, both chemogenetics and optogenetics have drawbacks that must be taken into account (see Table 1). The most relevant difficulty, with either optogenetics or chemogenetics, is to induce a neuronal or astrocytic activity that could mimic that observed under physiological conditions. Furthermore, ChR2 is permeable to Na^+^, K^+^and H^+^ (Nagel et al., 2003). Accordingly, ChR2 openings strongly depolarizes astrocytic cell membrane and alters internal pH and Na^+^ content. These effects, that have been involved in ChR2-mediated glutamate release from astrocytes (Sasaki et al., 2012; Beppu et al., 2014), may also interfere with astrocyte physiology. Another issue is the gene delivery of opsins or DREADDs to astrocytes which is commonly achieved with viral vectors, a technique that may also alter astrocyte physiological functions by activating an inflammatory response and gliosis. Furthermore, while optogenetics can be used not only to stimulate, but also to inhibit neurons (a very useful approach when interrogating neuronal networks, especially in awake animals) an effective inhibition of astrocyte Ca^2+^ signaling by optogenetic tools has been not achieved yet. Inhibition of astrocyte Ca^2+^ signal can be obtained through BAPTA-mediated Ca^2+^ chelation (Gómez-Gonzalo et al., 2010), by depleting intracellular Ca^2+^ stores with sarco/endoplasmic recticulum Ca^2+^ ATPase (SERCA) pump inhibitors, such as cyclopiazonic acid (CPA), or by using a transgenic mouse lacking IP3 receptors in astrocytes (Srinivasan et al., 2015). Alternatively, expression of Pleckstrin Homology (PH) domain of Phospholipase C (PLC)-like protein p130 (p130PH) via viral vectors was used to reduce Ca^2+^ signaling in astrocytes *in vivo* (Xie et al., 2010). While all these approaches provided important information on astrocyte functions, they are limited by drawbacks (Serrano et al., 2006; Jourdain et al., 2007; Gómez-Gonzalo et al., 2010). Accordingly, although the development of novel tools to obtain *on demand* rapid and effective silencing of astrocyte Ca^2+^ signals is highly desired, such implements have yet to come.

**Table 1:** **New tools to study Ca^2+^ dynamics in astrocytes**.

	Advantages	Disadvantages
**Imaging techniques**
2-photon and 3-photon microscopy	Recording from cortical and subcortical astrocytes (1 mm depth) in awake animals (Horton et al., 2013; Perea et al., 2014).	Imaging on deep structures (>1 mm depth) not feasible.
Optical microendoscopes or microprisms	Imaging of deep brain (>1 mm depth) areas (Chia and Levene, 2009).	Invasive, potential damage and inflammation of the imaged brain region.
**Ca^2+^ sensitive dyes**
Genetically-Encoded calcium indicators (GECIs)	Ca^2+^ activity at the distal fine astrocytic processes can be monitored (Srinivasan et al., 2015). Several tools (cre-lox system, viral vectors, *in utero* electroporation) to express GECIs in specific brain areas (Paukert et al., 2014; Gee et al., 2015; Srinivasan et al., 2015).	Several factors (expression techniques, promoter sequences) are critical for a good signal to noise signal. Potential cytotoxicity.
**Manipulation of astrocyte activity**	
Chemogenetics (DREADD, KORD)	Specific activation mediated by designed drug (Urban and Roth, 2015). Receptor activation takes several minutes and can last for hours (Guettier et al., 2009; Vardy et al., 2015).	Not suitable for fast astrocytic activation. DREADDs inhibiting astrocytic Ca^2+^ activity are still lacking.
Optogenetic (ChR2, Arch)	Fast Ca^2+^ increase through opsins (Deisseroth, 2015). Optogenetic astrocyte activation induces gliotransmitter release (Ji and Wang, 2015).	Optogenetic evoked Ca^2+^ dynamics differ from physiological activity recorded in astrocytes. Optogenetic stimulation can alter internal pH eliciting unknown effects (Nagel et al., 2003). On demand inhibition of astrocyte Ca^2+^ activity still lacking.

## Conclusions

Almost a century of neuron-centric research has left a deep gap in our knowledge of astrocyte physiology. The role of astrocytes in brain function and dysfunction emerged as a major topic in neuroscience in the last decade after the recognition of their dynamic modulation of synaptic functions and the evidence of their involvement in the early stages of neurological disorders, including epilepsy, ischemia, Alzheimer’s and Parkinson’s diseases (Barres, 2008; Allaman et al., 2011; Losi et al., 2012; Pekny et al., 2016) making astrocytes attractive targets for novel therapeutic strategies. The study of the role of astrocytes in the brain requires specifically designed experimental approaches due to the distinct morphology and the unique functional properties of astrocytes. Tailoring novel molecular tools for astrocytes, such as chemogenetics and optogenetics, combined with the most advanced Ca^2+^ imaging techniques *in vivo*, will allow greater understanding of astrocyte functions in the brain.

## Author Contributions

GL and GC wrote the abstract, introduction, selective tools to study neuron-astrocyte cross-talk, conclusion and supervised the manuscript. LM wrote the paragraph on Imaging techniques. MS wrote the paragraph on calcium sensitive dyes and Table 1.

## Conflict of Interest Statement

The authors declare that the research was conducted in the absence of any commercial or financial relationships that could be construed as a potential conflict of interest.

## Abbreviations

CNO: clozapine-N-oxide
DREADDs: designer receptors exclusively activated by designer drugs
GECI: genetically encoded calcium indicators
GFAP: glial fibrillary acidic protein
GLAST: glutamate aspartate transporter
ROI: region of interest
S100β: S100 calcium-binding protein B
SERCA: sarco/endoplasmic recticulum Ca^2+^ ATPase.
